# The “Undefined and Ignored Normal Tissue” Bulboclitoral Complex in Locally Advanced Cervical Cancer Treated with Definitive Radiochemotherapy: Is It Not the Organ at Risk?

**DOI:** 10.3390/medicina62010014

**Published:** 2025-12-21

**Authors:** Kamuran Ibis, Mahmut Hudai Aydin, Korhan Kokce, Leyla Suncak, Ozlem Guler Guniken, Can Ilgin, Deniz Bolukbas, Nezihe Seden Kucucuk, Inci Kizildag Yirgin

**Affiliations:** 1 Department of Radiation Oncology, Institute of Oncology, Istanbul University, 34093 Istanbul, Türkiye; aydinhudai@istanbul.edu.tr (M.H.A.); korhan.kokce@istanbul.edu.tr (K.K.); ozlem.guler@istanbul.edu.tr (O.G.G.); can.ilgin@istanbul.edu.tr (C.I.); deniz.bolukbas@istanbul.edu.tr (D.B.); nezihe.kucucuk@istanbul.edu.tr (N.S.K.); 2 Department of Medical Physics, Institute of Oncology, Istanbul University, 34093 Istanbul, Türkiye; leyla.suncak@istanbul.edu.tr; 3 Department of Radiology, Institute of Oncology, Istanbul University, 34093 Istanbul, Türkiye; inci.kizildagyirgin@istanbul.edu.tr

**Keywords:** clitoris, radiotherapy, uterine cervical neoplasms, gynecology, women, sexual health, vagina

## Abstract

*Background and Objectives:* The bulboclitoral complex (BCC) is an essential organ for female sexual health. However, it is not defined as an organ at risk in any guideline defining target volumes in radiotherapy of gynecological cancers, and there is no information about dose constraint. *Materials and Methods:* Simulation computed tomography scans of 20 patients diagnosed with locally advanced cervical cancer were used retrospectively. The volumetric modulated arc therapy treatment plan with a total dose of 45 Gy in 25 fractions was created using the planning target volume (PTV)-standard, which was created without considering the BCC, and the PTV-BCC spared, which were contoured and included in the optimization. Bulboclitoral complex doses in PTV-standard and PTV-BCC spared plans were compared using the paired simple *t* test. *Results*: Median BCC volume was 17.6 cm^3^ (11.20–25.50). Bulboclitoral complex maximum dose (Dmax) was median 49.07 Gy (48.49–50.25) and 28.81 Gy (18.14–44.61) in the PTV-standard and PTV-BCC spared plans, respectively, and the BCC Dmax was statistically significantly lower in the PTV-BCC spared plan (*p* < 0.001). When comparing BCC percentage of volume receiving 45 Gy (V45), the median values for PTV-standard and PTV-BCC spared plans were 37.5% (13.3–82.6) and 0%, respectively (*p* ≤ 0.001). *Conclusions*: The bulboclitoral complex can be dosimetrically protected from radiation by contouring and optimizing it as an organ at risk in the radiotherapy plan. The clinical effects of protecting the BCC from radiation as an organ at risk on sexual health need to be investigated.

## 1. Introduction

Cervical cancer is the third most common malignant tumor in women worldwide, both in terms of incidence and mortality [1]. In locally advanced cervical cancer (LACC), adaptive brachytherapy (3D-IGABT) guided by magnetic resonance imaging (MRI)-based three-dimensional imaging is the preferred treatment method following chemoradiotherapy (CRT) [2]. An increasing number of cervical cancer patients, predominantly young or middle-aged women, may experience long-term bowel and bladder symptoms, fatigue, and sexual problems that can negatively impact their health-related quality of life [3,4,5]. Sexual issues experienced by survivors include problems with sexual desire, arousal, pleasure, satisfaction, and vaginal function during sexual intercourse or other sexual activities [4,5,6,7,8].

The bulboclitoral complex (BCC), consisting of the vestibular and clitoral erectile tissues, forms the anatomical basis of female sexual arousal and orgasm [9,10,11]. In 2005, O’Connell et al. provided the most comprehensive description of the anatomy of the clitoris, including its components, vascular nerves, and adjacent structures, based on histological and immunohistochemical studies, as well as MRI and three-dimensional cross-sectional anatomical reconstructions [10,12].

In the atlas published by Brooks et al., the external genital organs are defined as organs at risk in anal cancer radiotherapy (RT), including the skin, fat, and a small portion of the BCC [13]. Marshal and colleagues developed a contouring guide for RT in their organ preserving feasibility studies, which identified the BCC as an organ at risk in 20 patients treated for anal canal cancer [9,14]. Although the effects of RT on organs vital for sexual and reproductive function in female patients, such as the vagina or ovaries, have been extensively studied, the impact of RT on erectile tissues remains poorly understood [3,4,7].

In the literature, information about the anatomy of the BCC and the effects of radio-therapy on the BCC in female patients treated with RT is limited. In our study, we aimed to investigate the relationship between BCC, which we believe should be considered as an essential erectile tissue despite its unknown radiological anatomy, and the treatment field in curative cervical cancer contouring and planning, by examining the doses it receives. This was done to remove it from the category of “unidentified and ignored normal tissue,” thereby increasing the knowledge of radiation oncologists and drawing attention to its evaluation as an organ at risk.

## 2. Materials and Methods

**Figure 1:**
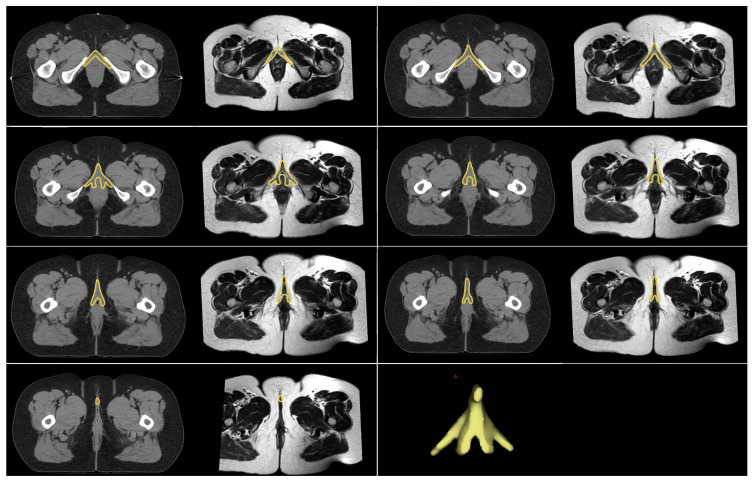
In each slice, on the left, the contour of the BCC on the simulation CT, on the right, the contour of the BCC on the T2-weighted magnetic resonance imaging, and the yellow shape on the bottom right, the BCC. (CT, Computed tomography; BCC, Bulboclitoral complex).

Simulation computed tomography (CT) scans of 20 patients diagnosed with locally advanced cervical cancer who underwent curative CRT followed by brachytherapy were included in this dosimetric study. Simulation CT scans were taken from the patients with a full bladder and an empty bladder at 3 mm slice thickness using the Siemens SOMATOM go.sim CT Simulator device (Siemens Healthineers AG, Forchheim, Germany). These simulation CT scans were copied into the Varian Eclipse v17.0.1 treatment planning system (Varian Medical Systems, Palo Alto, CA, USA). All organs at risk and target volumes were re-contoured. Two PTVs were planned to be created: PTV-standard and PTV-BCC spared. The CTV volume was defined to include the common iliac lymph nodes at the superior border and the entire vagina at the inferior border, assuming the presence of negative lymph nodes. Before contouring the BCC, the radiation oncologist contoured the CTVs on simulated CT scans taken with both a full and empty bladder to create the internal target volume (ITV). A 1 cm margin was added to this ITV to create the PTV and named it PTV-standard. Rigid fusion was then performed using a T2-weighted MRI sequence and simulated CT images. Considering the anatomic location of the BCC, the pubic bones were used as reference points during the fusion process. The BCC was contoured by a radiologist experienced in pelvic MRI (Figure 1). To create PTV-BCC spared, PTV-standard was copied and only the distal border was adjusted. During this adjustment, care was taken to ensure that the PTV did not overlap the BCC volume. To ensure a standardized evaluation, the lower border of the PTV-BCC spared was passed through the lower border of the symphysis pubis, taking into account the anatomy of the BCC.

Treatment plans were created using the Anisotropic Analytic Algorithm (AAA) algorithm in the Eclipse v17.0.1 treatment planning system with a 3-field volumetric modulated arc therapy (VMAT) planning technique (Varian Medical Systems, Palo Alto, CA, USA). When planning PTV-BCC spared, it was included in the optimization to preserve the BCC. When BCC is included in optimization, the goal is for BCC to receive the least possible dose without compromising the target OAR doses and target doses in the treatment plan using PTV-standard. The treatment plan was recreated without changing other optimization criteria.

In the treatment plan, 30% of the bowel is <40 Gy, 35% of the bladder is <45 Gy, 55% of the bladder is <55 Gy, 15% of the femoral head is < 30–35 Gy, femur head 5% < 50 Gy, rectum 60% < 30–35 Gy, rectum 50% < 50 Gy, bilateral kidney mean < 15–18 Gy usual tissue constraints were used. The treatment plan was designed to deliver 45 Gy in 25 fractions, with 95% of the dose reaching at least 95% of both PTVs. A total of 40 VMAT treatment plans were created using PTV-standard and PTV-BCC spared target volumes.

The quality of the radiation therapy was evaluated by two indices calculated according to these two formulae: Homogeneity index (HI): (D2–D98%)/D50% and conformity index (CI): The volume covered by 95% of the isodose lines (VRI)/PTV [15].

Bulboclitoral complex doses obtained from PTV-standard and PTV-BCC spared plans were compared using a Paired Simple T-test. Statistical analysis was performed using Statistical Package for Social Sciences for Windows software version 22 (IBM Corp, Armonk, NY, USA). *p* < 0.05 was considered statistically significant.

This study was approved by the Clinical Research Ethics Committee of Istanbul Faculty of Medicine, Istanbul University (Approval Number: 2024/1305, Date: 12 July 2024).

## 3. Results

**Figure 2:**
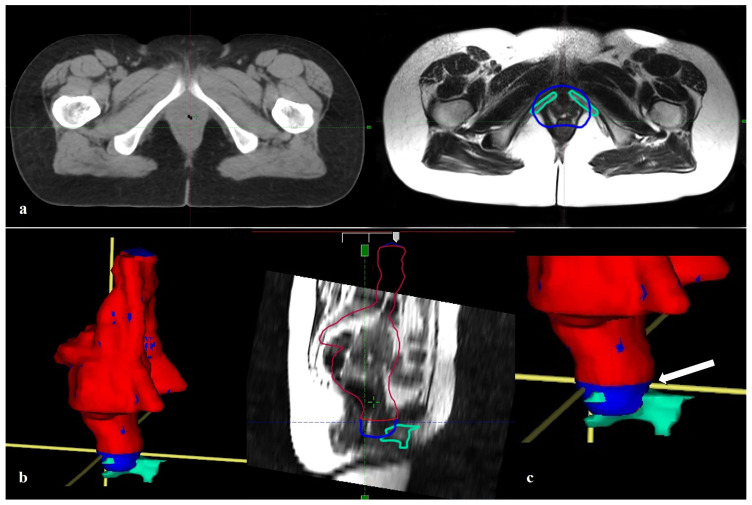
(**a**) Simulation CT on the left, axial section through the lower border of the pubic symphysis on magnetic resonance imaging on the right. (**b**) Red-colored PTV-BCC spared ending at the lower border of the pubic symphysis on the left, blue-colored PTV-standard extending down from the lower border of the pubic symphysis surrounding the BCC in the distal region. In the middle, contour views of the PTV and the BCC on magnetic resonance imaging. (**c**) Close-up view of the relationship between the PTVs and the BCC (white arrow points to the lower border of the pubic symphysis). (CT, Computed tomography; PTV, Planning target volume; BCC, Bulboclitoral complex).

Median BCC volume was 17.6 cm^3^ (range, 11.20–25.50). The median volume of PTV-standard was 1437.9 cm^3^ (range, 1058.8–1926.3), and the median volume of PTV-BCC spared was 1406 cm^3^ (range, 1019.8–1898.9). The difference between PTV-standard and PTV-BCC spared volumes was median 32.6 cm^3^ (range, 16.3–48.9), which was statistically significant (*p* < 0.001). Figure 2 illustrates the PTV-standard and the PTV-BCC spared volumes about the BCC and the lower border of the symphysis pubis.

Organ dose limits were within acceptable limits for both plans (Table 1). The HI values for PTV-standard and PTV-BCC spared plans were median 0.09 (range, 0.08–0.11) and median 0.09 (range, 0.09–0.1), respectively. The CI values for the PTV-standard and PTV-BCC spared plans are median 1 (range, 0.95–1.05) and median 1 (range, 0.96–1.07), respectively.

Bulboclitoral complex dose maksimum (Dmax) in PTV-standard and PTV-BCC spared plans are median 49.1 Gy (range, 48.5–50.3) and median 28.8 Gy (range, 18–44.6), and the BCC Dmax dose was significantly lower in the PTV-BCC spared plan (*p* < 0.001). The BCC minimum dose (Dmin) was median 6.6 Gy (range, 3.3–16.6) and median 3.2 Gy (range, 2.2–6), and the BCC Dmin dose is significantly lower in the PTV-BCC spared plan (*p* < 0.001).

**Figure 3:**
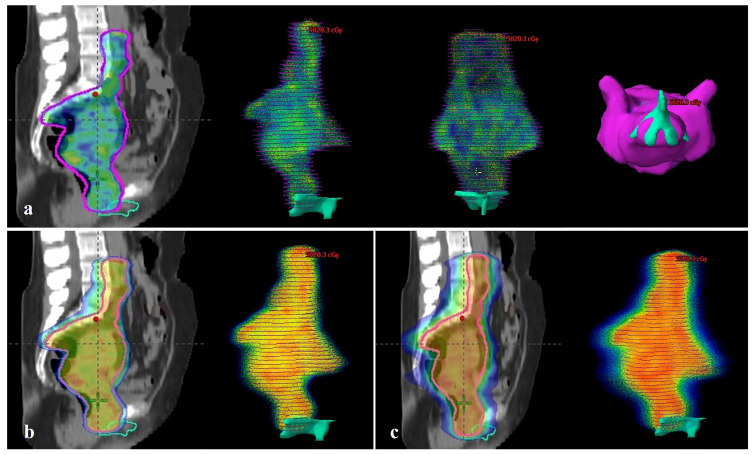
PTV-standard treatment plan (**a**) On the left, the 45 Gy dose distribution on simulation CT; on the right, the 45 Gy dose distribution and its relationship with the BCC, respectively, in lateral, coronal, and food images (median V45 = 37.5%, the 45 Gy dose appears to surround the BCC); (**b**) On the left, the 35 Gy dose distribution on simulation CT; on the right, the 35 Gy dose distribution and its relationship with the BCC (median V35 = 63.9%); (**c**) On the left, the 25 Gy dose distribution on simulation CT; on the right, the 25 Gy dose distribution and its relationship with the BCC (median V25 = 81.1%). (PTV, Planning target volume; CT, Computed tomography; BCC, Bulboclitoral complex).

**Figure 4:**
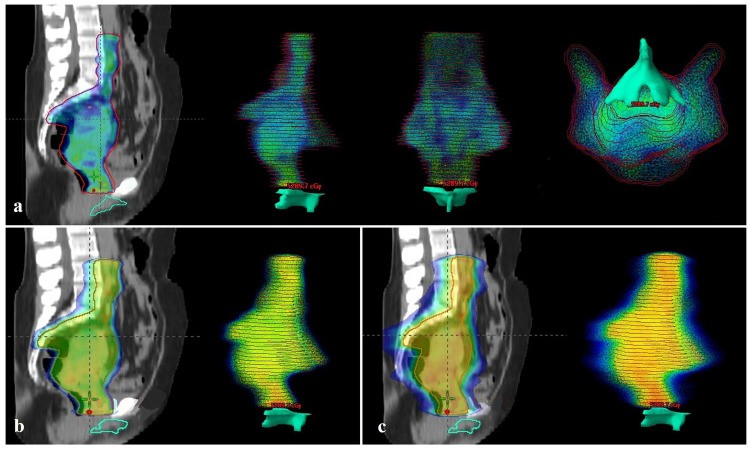
PTV-BCC spared treatment plan. (**a**) On the left, the 45 Gy dose distribution in simulation CT; on the right, the 45 Gy dose distribution and its relationship with the BCC, respectively, in lateral, coronal, and axial images (median V45 = 0; the 45 Gy dose appears to end above the BCC). (**b**) On the left, the 35 Gy dose distribution on simulation CT; on the right, the 35 Gy dose distribution and its relationship with the BCC (median V35 = 0). (**c**) On the left, the 25 Gy dose distribution in simulation CT scan; on the right, the 25 Gy dose distribution and its relationship with the BCC (median V25 = 0.18%). (PTV, Planning target volume; CT, Computed tomography; BCC, Bulboclitoral complex).

When compared to BCC volume receiving 45 Gy (V45), the median values for PTV-standard and PTV-BCC spared plans were 37.5% (range, 13.3–82.6) and 0%, respectively (Figure 3a and Figure 4a, respectively), statistically significantly higher in the PTV-standard plan (*p* < 0.001). When comparing BCC V40, the median values were 53.6% (range, 26.5–88.4) and 0% (range, 0–0.71) for PTV-standard and PTV-BCC spared plans, respectively; the BCC volume receiving 40 Gy was statistically significantly higher in the PTV-standard plan (*p* < 0.001). When compared to BCC V35, the median values for PTV-standard and PTV-BCC spared plans were 63.9% (range, 46.6–92.5) and 0 (range, 0–1.77), respectively (Figure 3b and Figure 4b, respectively); statistically significantly higher in the PTV-standard plan (*p* < 0.001) (Figure 3b and Figure 4b, respectively).

When comparing BCC V30, the median values for PTV-standard and PTV-BCC spared plans are 72.5% (range, 49.3–96.3) and 0% (range, 0–3.07), respectively, statistically significantly higher in the PTV-standard plan (*p* < 0.001). When compared to BCC V25, the median values for PTV-standard and PTV-BCC spared plans were 81.1% (range, 58.8–98.8) and 0.18% (range, 0–4.8), respectively (Figure 3c and Figure 4c, respectively). The BCC volume receiving 25 Gy is significantly higher in the PTV-standard plan (*p* < 0.001) (Figure 3c and Figure 4c, respectively). When comparing BCC V20, V15, V10, and V5, the BCC volumes receiving 20 Gy, 15 Gy, 10 Gy, and 5 Gy in the PTV-standard plan were also higher (*p* < 0.001) (Table 2).

## 4. Discussion

In our study, median BCC volume was 17.6 cm^3^ (11.20–25.50). When the BCC is contoured and included in the treatment plan as an organ at risk, the maximum radiation dose it receives decreases statistically significantly (median 49.1 Gy vs. 28.8 Gy, *p* < 0.001). Thus, the BCC volume receiving 45 Gy, 40 Gy, and 35 Gy radiation doses decreased from the median 37.5% to 0, 53.6% to 0, and 63.9% to 0, respectively. Similar differences were seen in BCC volumes receiving 30 Gy, 25 Gy, 20 Gy, 10 Gy, and 5 Gy radiation doses. Organ dose limits were within acceptable limits for both plans.

Bulboclitoral erectile tissue is an essential structure in the pelvic region that plays a significant role in sexual function. However, while the effects of pelvic RT on sexual dysfunction in gynecological malignancies have been studied, the focus has primarily been on its impact on vaginal function. Pathological changes associated with RT include reduced vaginal epithelial volume, increased distance between dermal papillae, decreased distance from the basal layer to the epidermis, and mucosal atrophy [16]. Acute and chronic side effects include vaginal dryness, dyspareunia, decreased sexual desire, loss of vaginal elasticity, vaginal fibrosis, and stenosis, a permanent vaginal change caused by RT, which may lead to sexual dysfunction, causing discomfort during intercourse [17].

In a prospective analysis of sexual outcomes from the EMBRACE study, crude incidence rates of 38.4% for vaginal dryness and 33.5% for pain during intercourse were reported in patients with LACC treated with CRT and IGABT. In long-term analysis, patients reported a slight improvement in pain during sexual intercourse over time, along with an increase in sexual activity, but no improvement in vaginal dryness over time. Additionally, vaginal shortening and narrowing were recorded in 34–36% of patients due to ongoing increases in toxicity. Due to these changes, 37–48% of patients reported experiencing “no” or “some” sexual pleasure [18]. We believe that side effects on the vaginal structure are an important component of sexual health, but the effects of RT on BCC, which has a primary role in sexual pleasure and orgasm, should be investigated.

Westerveld et al. investigated the validation of the PIBS (posterior-inferior border of the symphysis) vaginal dose reporting method. They evaluated the dose-effect relationship between vaginal dose points and vaginal stenosis in a prospective patient cohort. The study included 301 patients with a median follow-up of 49 months. The incidence of vaginal stenosis was 25%, 52%, 20%, and 3%, respectively, for grades 0, 1, 2, and 3. The median total doses for the PIBS + 2 cm, PIBS, PIBS − 2 cm, and ICRU-RV reference points were 52.9, 41, 4.1, and 64.6 Gy EQD2_3Gy_, respectively. Vaginal involvement at diagnosis, advanced age, shorter VRL (vaginal reference length), and the use of a tandem-ovoid applicator were identified as risk factors for vaginal stenosis. Most importantly, they reported that higher doses applied to the PIBS, PIBS + 2 cm, and RV reference points were significantly associated with an increased risk of vaginal stenosis grade ≥ 2 [19].

**Figure 5:**
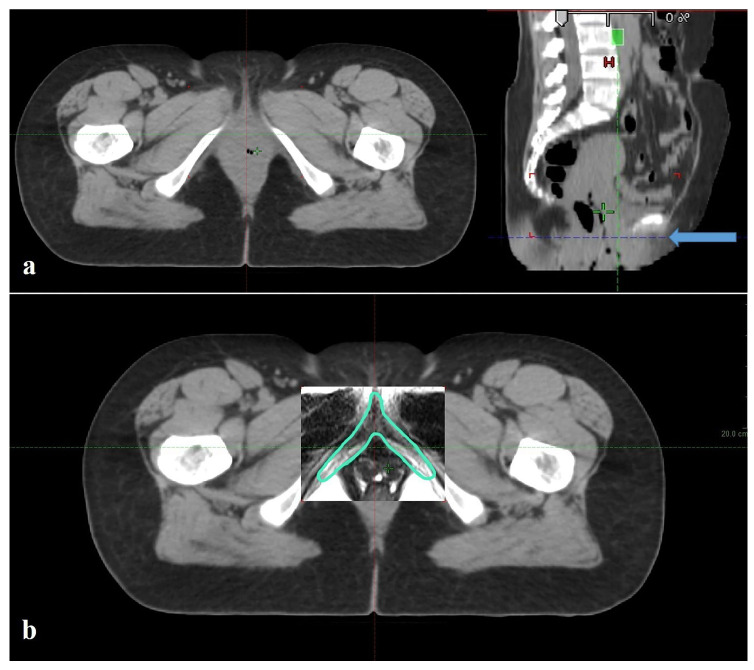
(**a**) Distal vaginal image on axial simulation CT section passing through the PIBS. Radiological anatomy cannot be distinguished (Blue arrow points to the inferior border of the PIBS in the sagittal CT section on the right). (**b**) When simulation CT is fused with T2-weighted magnetic resonance imaging, structures in the same section and the BCC, which we did not see, can be easily identified (Green shape is BCC). (CT, Computed tomography; PIBS, Inferior border of the symphysis pubis; BCC, Bulboclitoral complex).

The BCC typically begins at the PIBS level (Figure 5), so the PIBS dose may not be the only factor necessary for vaginal toxicity. The BCC should be contoured and evaluated in both planning and toxicity studies. In our study, we limited the lower boundary of the target volume in the PTV-BCC spared plan to the PIBS level. When we included this volume and the BCC in optimization, we demonstrated that we could preserve the BCC and that the PIBS level could serve as an essential reference.

In the 2008 article by Small et al., titled “Consensus guidelines for delineation of clinical target volume for intensity-modulated pelvic RT in postoperative treatment of endometrial and cervical cancer,” the vaginal CTV definition should include the upper 3 cm of the vagina and the paravaginal soft tissue lateral to the vagina. The lower boundary is defined as extending 3 cm below the upper boundary of the vagina or 1 cm above the lower boundary of the obturator foramen, whichever is lower. There is no PTV recommendation in the study. The text does not mention the clitoris. We recommend paying attention to the proximity of the lower boundary to the PIBS level and its relationship with BCC when creating the PTV [20].

In the 2011 article by Lim et al., “Consensus guidelines for delineation of clinical target volume for intensity modulated pelvic RT for the definitive treatment of cervix cancer,” it is recommended that: In tumors with small or no vaginal fornix involvement, the upper half of the vagina should be included in the CTV; in tumors with upper vaginal involvement, the upper two-thirds of the vagina should be treated; and in tumors with the extensive participation, the entire vagina should be included in the CTV. The vulva and perineum should not be included in the CTV unless they are significantly involved. For PTV creation, if high-quality daily soft tissue verification is available during treatment, margins of 1.5 to 2 cm are recommended around the CTV. The article suggests using diagnostic MRI or MRI simulation scans for contouring, and fusion of T2-weighted axial MRI images with the planning CT. In the study, GTV, CTV, and OARs are presented in detail and in an instructive manner on MRI [21]. BCC is not mentioned. BCC is easily visible on T2-weighted MRI. Additionally, while the uterus and cervix are highly mobile structures, BCC can be accurately contoured by using the pubic bones as a reference and performing MRI fusion with simulation CT. CTVs vary depending on the level of vaginal involvement. When a margin is added to create the PTV, the distal vaginal border will approach or extend below the PIBS. Sometimes the lower vaginal margin is lowered not because of vaginal involvement but due to a bulky exophytic tumor. At this point, we recommend that radiation oncologists evaluate treatment volumes based on patient risk and treat the target while preserving erectile tissue whenever possible.

In their 2012 article, “Pelvic normal tissue contouring guidelines for radiation therapy: A Radiation Therapy Oncology Group Consensus,” Gay et al. defined the normal pelvic tissues in men and women as the rectum, anus + rectum, sigmoid, bowel bag, small bowel, colon, anus + rectum + rectosigmoid, mesorectum, bladder, uterus + cervix, ovaries + fallopian tubes, prostate, seminal vesicles, penile bulb, and proximal femurs. The clitoris is not mentioned at all, while the vagina is only referenced in the statement “Including the anus is most relevant when treating the distal vagina or vulva.” The vagina and BCC are not defined as normal female pelvic tissue [22]. Vaginal toxicity has been studied in detail in pelvic RT for gynecological cancers and continues to be studied; BCC requires the necessary attention. Additionally, care must be taken to protect these organs from side effects in all diseases treated with pelvic irradiation.

Tanderup et al. presented the EMBRACE II protocol version 1.0 on 15 October 2015, which is an excellent protocol used worldwide by radiation oncologists for the treatment of cervical cancer, including external RT and 3D-IGABT, as well as treatment side effects. They have conducted groundbreaking studies in the treatment of cervical cancer and the management of side effects and continue to do so. The protocol does not mention the clitoris. Detailed studies have been conducted on vaginal toxicity, and essential points have been highlighted. It is recommended that PIBS and PIBS ± 2 cm point doses be measured in both external RT and brachytherapy [23]. In 2016, the International Commission on Radiation Units and Measurements & Groupe Européen de Curietherapie—European Society for Radiotherapy and Oncology (ICRU & GEC—ESTRO) published ICRU Report No. 89, Prescribing, Recording, and Reporting Brachytherapy for Cancer of The Cervix, under the heading “Recommendations for Reporting.” In the table “Research-oriented reporting, OAR volumes and points,” mentions “vulva (labia, clitoris).” The vagina is discussed in detail, with emphasis on PIBS points (PIBS, PIBS ± 2 cm) [24]. In our study, which aims to emphasize the localization, contouring, and evaluation of BCC as an OAR in treatment planning, we demonstrated that the PIBS level is a critical threshold. We believe that the PIBS level is of vital importance for BCC.

In the 2016 guidelines titled “Consensus recommendations for radiation therapy contouring and treatment of vulvar carcinoma” by Gaffney et al., the clitoris is mentioned in two places. First, as one of the sites of origin of vulvar cancer; second, when stating that the suspensory ligament of the clitoris should also be included in the CTV in cases of clitoral lesions. Only the external genital portion of the clitoris is mentioned [25]. However, when the contour volume is examined, it is observed that the majority of BCC is included within the volume.

Small et al. published the “NRG Oncology/RTOG consensus guidelines for delineation of clinical target volume for intensity-modulated pelvic radiation therapy in postoperative treatment of endometrial and cervical cancer: An update” in 2020. The guidelines do not mention the clitoris. The vaginal CTV is defined as “Approximately 3.5 to 4 cm of the proximal vaginal canal measured from the apex should be included in the vaginal CTV. For patients with extensive LVSI, positive vaginal margin, or adverse pathology, a longer length of the vagina may be treated.” If ITV is used in conjunction with daily cone-beam CT imaging guidance, a narrower vaginal PTV margin of 6 to 8 mm may be considered a safe option. If ITV is not used and imaging guidance is not used or used minimally, a margin of 1.5 to 2.0 cm or larger is recommended [26]. We recommend that radiation oncologists evaluate treatment volumes based on patient risk and treat the target that requires treatment while preserving erectile tissue when necessary.

Schmid et al. (2020) [27] in their guideline “Recommendations from gynaecological (GYN) GEC-ESTRO working group—ACROP: Target concept for image-guided adaptive brachytherapy in primary vaginal cancer” mention the clitoris only in the sentence “In particular, in tumors involving the lower third of the vagina, additional anatomical structures such as the vulva and clitoris should be considered. For all these organs, the outer wall or boundary should be contoured.”. Venkatesh et al. evaluated the feasibility of creating a BCC contour and the dose received by the BCC in patients who underwent interstitial gynecological brachytherapy for tumors involving the lower vagina and periurethral region in a retrospective cohort study. A total of 5 patients received IMRT external beam radiation therapy (EBRT) (45 Gy in 25 fractions), followed by HDR Ir-192 interstitial brachytherapy using the CT/MR Mick-Alektiar-Cohen interstitial gynecology template for a total dose of 25 Gy (range, 22.5–27.5 Gy) in 5 fractions. BCC was retrospectively contoured using T2-weighted MRI sequences combined with pre-treatment and brachytherapy CT simulations. The average IMRT dose to the BCC was 45.87 Gy (range 44.79–46.66 Gy), and the average HDR dose was 14.02 Gy (range 11.23–18.88 Gy). Assuming an alpha-beta ratio of 3 Gy, the average bulboclitoral D90 EQD2 was 62.93 Gy (range 58.72–67.22 Gy). In the acute phase, severe pain in the clitoral glans region was reported in all patients, and in one patient, decreased clitoral sensitivity and inability to achieve clitoral orgasm were reported 5 months after RT [28]. We recommend contouring the BCC, especially if the target volume approaches the PIBS level.

In the 2024 guidelines titled “Consensus guidelines for delineation of clinical target volumes for intensity modulated radiation therapy for intact cervical cancer: An update” by Fields et al., one of the three cases selected showed a vaginal drop metastasis in the lower 1/3 of the vagina on the right lateral wall. No topographic boundary was specified for the lower vaginal margin, with an emphasis on “enlarging to ensure a margin on vaginal disease.” Additionally, it is recommended that all CTVs be excluded from muscle and bone [29]. No recommendation is provided regarding the clitoris. We believe that BCC should not be included in the CTV unless there is invasion, and if it remains within the treatment target area, the dose it receives should be calculated.

The EMBRACE-vaginal morbidity substudy prospectively evaluated vaginal changes assessed by a physician, and patient-reported vaginal and sexual function problems and distress outcomes in the first 2 years after image-guided radio(chemotherapy)therapy and brachytherapy for LACC. In the study, which included 113 eligible patients, mild (Grade 1) vaginal changes were detected in 20% of patients (range 11–37%). At 2 years, 47% were not sexually active. Approximately 50% of sexually active women reported any vaginal and sexual function problems and distress over time; more significant vaginal and sexual issues and distress were reported in 14%, 20%, and 8% of patients, respectively. Vaginal changes assessed by a doctor and patient-reported outcomes (PRO) for sexual satisfaction showed significant differences between baseline and the first follow-up, without further substantial changes over time (*p* ≤ 0.05). No association was found between physician-assessed vaginal changes and PRO vaginal function problems and sexual distress [30]. Suvaal et al. reported that mild vaginal changes were likely due to a combination of tumors with limited vaginal involvement, EMBRACE-specific treatment optimization, and rehabilitation recommendations. Researchers noted that the absence of an association between vaginal changes, vaginal function problems, and sexual distress indicates that sexual function is more complex than vaginal morbidity alone [30]. In this context, we believe that awareness of BCC among women, as well as its contouring and protection as an OAR in treatment planning, is essential.

Our study is the first to evaluate the BCC as an organ at risk in definitive pelvic RT for cervical cancer. It emphasizes the importance of radiation protection for erectile tissue in women, an important target in RT-induced sexual dysfunction, which has not been studied before. It is clear that current guidelines lack specific recommendations regarding the importance and contouring of BCC in sexual function. Standardized recommendations on this topic would foster international unity and momentum for radiation protection of BCC and the assessment of its effects on sexual health.

One of the limitations of our study is that the lower limit of one of the compared target volumes was limited to the PIBS, while the other extended below the PIBS. However, our aim in this selection was to reflect the results of target volume contouring performed without considering the presence of BCC. In clinical practice, the lower limit of the CTV and the planned target volume vary depending on vaginal involvement when creating the target volume, and differences arise depending on the treating physician. For these reasons, we selected treatment volumes that demonstrated that we could preserve BCC in appropriate patients and that provided a fixed point. Another limitation is the small cohort size and the lack of patient-reported outcomes or sexual function data. Our study is a dosimetric study designed to highlight the importance of considering BCC contouring and radiotherapy plan optimization in treatment plans for patients with cervical cancer undergoing curative CRT. Further studies examining clinical data, sexual function, and patient-reported outcomes are needed.

Another limitation of our study is that it only included external radiotherapy planned using the VMAT technique. Our study focused on contouring the bulboclitoral complex and protecting it by incorporating it into treatment optimization, so we used the VMAT technique because it is standard practice. Studies using different techniques can also be conducted for the same purpose; determining which technique is more successful may be a question for a separate study. Furthermore, one of the limitations of our study is the lack of assessment of the doses received by the BCC during brachytherapy, a crucial component of curative cervical cancer treatment. Due to the dosimetric nature of our study and the lack of patient data on sexual function, we did not evaluate the brachytherapy plans. However, because the anatomic location of the BCC is at or below the PIBS and because it is designated as a reference point for vaginal toxicity assessment in the EMBRACE studies and ICRU 89, we considered the PIBS as the critical point in our study [23,24].

While tumor location is the critical parameter in determining target volume, and disease control is the primary goal, erectile tissue preservation should be considered in patients where BCC can be preserved without compromising tumor control. We acknowledge the lack of substantial evidence to support this recommendation. However, we ask the following question: Why should erectile tissue not be protected from the effects of radiotherapy? The BCC is a key structure in the pelvic region that plays a significant role in sexual function. Our goal should be to raise awareness, provide education, and conduct research to evaluate the dose response of BCC and preserve sexual function. Clinical studies are needed to evaluate the effect on sexual function and the dose–response relationship according to the radiation dose received by the BCC.

## 5. Conclusions

The BCC is erectile tissue which is not identified as an organ at risk in pelvic RT treatment plans, is overlooked when focusing on vaginal side effects in the evaluation of treatment-related sexual side effects, and can receive a significant radiation dose depending on the target volume. The dose increases when the treatment area extends to the lower border of the symphysis pubis. Learning the radiological anatomy of this erectile tissue, which can be easily distinguished on T2-weighted MRI; incorporating it into optimization of treatment plans; evaluating it for sexual side effects; and investigating the effects of radiation are rational and scientific duties and responsibilities.

## Figures and Tables

**Table 1:** Organ-at-risk doses obtained from both treatment plans.

	PTV-Standard Median Min–Max	PTV-BCC Spared Median Min–Max
Bowel 30% (Gy)	21 17.57–25.65	23.09 18.28–26.16
Bladder 35% (Gy)	43.27 39.65–45.67	44.13 41.03–45.78
Bladder 55% (Gy)	37.93 31.17–41.63	38.38 31.86–42.32
R-femoral head 15% (Gy)	24.24 19.61–26.41	25.59 17.82–28.13
R-femoral head 5% (Gy)	30.28 26.41–34.54	32.16 27.12–35.25
L-femoral head 15% (Gy)	24.36 17.88–27.61	23.90 16.19–26.73
L-femoral head 5% (Gy)	24.36 25.16–35.77	30.77 22.35–34.62
Rectum 60% (Gy)	36.28 28.19–43.59	37.52 26.61–43.34
Rectum 50% (Gy)	39.49 33.03–44.58	40.1 33.5–44.5
R-kidney mean dose (Gy)	1.24 0.83–3.77	1.21 0.81–3.72
L-kidney mean dose (Gy)	1.13 0.82–2.48	1.11 0.78–2.47
HI	0.09 0.08–0.11	0.09 0.09–0.1
CI	1 0.95–1.05	1 0.96–1.07

BCC, Bulboclitoral complex; Min, Minimum; Max, Maximum; Gy, Gray; R, Right; L, Left; HI, Homogeneity index; CI, Conformity index.

**Table 2:** Doses received by the clitoris in both treatment plans.

	PTV-Standard Median Min–Max	PTV-BCC Spared Median Min–Max	*p*-Value
PTV cm^3^	1437.9 1058.8–1926.3	1406 1019.8–1898.9	<0.001
BCC Dmax	49.1 48.5–50.3	28.8 18–44.6	<0.001
BCC Dmin	6.6 3.3–16.6	3.2 2.2–6	<0.001
BCC V45Gy (%)	37.5 13.3–82.6	0	<0.001
BCC V40Gy (%)	53.6 26.5–88.4	0 0–0.7	<0.001
BCC V35Gy (%)	63.9 46.6–92.5	0 0–1.8	<0.001
BCC V30Gy (%)	72.5 49.3–96.3	0 0–3.07	<0.001
BCC V25Gy (%)	81.1 58.8–98.8	0.18 0–4.8	<0.001
BCC V20Gy (%)	88.3 69.8–100	1.71 0–93	<0.001
BCC V15Gy (%)	93.5 78.5–100	5.2 0.08–13.7	<0.001
BCC V10Gy (%)	97.5 87.2–100	13.6 1.5–30.5	<0.001
BCC V5Gy (%)	100 96.8–100	65.5 40.3–91.1	<0.001

BCC, Bulboclitoral complex; PTV, Planning target volume; Dmax, Maximum dose; Dmin, Minimum dose; Gy, Gray; V45Gy, Percentage of volume receiving 45 Gy.

## Data Availability

The data presented in this study are available on request from the corresponding author due to ethical reasons.

## Abbreviations

The following abbreviations are used in this manuscript:

3D	Three-dimensional
BCC	Bulboclitoral complex
CI	Conformity index
CT	Computed tomography
CTV	Clinical target volume
Dmax	Maximum dose
Dmin	Minimum dose
EQD2	Equivalent dose of 2Gy
GEC—ESTRO	Groupe Européen de Curietherapie—European Society for Radiotherapy and Oncology
GTV	Gross tumor volume
Gy	Gray
HI	Homogeneity index
ICRU	International Commission on Radiation Units and Measurements
IGABT	Image-guided adaptive brachytherapy
ITV	Internal target volume
LACC	Locally advanced cervical cancer
MRI	Magnetic resonance imaging
PIBS	Posterior-inferior border of the symphysis
PTV	Planning target volume
RT	Radiotherapy
RV	Recto-vaginal
V45	Percentage of volume receiving 45 Gy
VMAT	Volumetric modulated arc therapy
VRI	The volume covered by 95% of the isodose line
VRL	Vaginal reference length
